# F68. PREMORBID IQ, EDUCATIONAL LEVEL AND JUMPING TO CONCLUSIONS AS PREDICTORS OF CLINICAL OUTCOME AT FIRST ONSET OF PSYCHOSIS OVER THE NEXT 4 YEARS: THE GAP STUDY

**DOI:** 10.1093/schbul/sby017.599

**Published:** 2018-04-01

**Authors:** Victoria Rodriguez, Olesya Ajnakina, Simona Stilo, Valeria Mondelli, Tiago Reis Marques, Antonella Trotta, Giada Tripoli, Diego Quattrone, Marco Colizzi, Poonam Sood, Ben Wiffen, Paola Dazzan, Evangelos Vassos, Marta Di Forti, Anthony David, Robin Murray

**Affiliations:** Institute of Psychiatry, Psychology & Neuroscience, King’s College London

## Abstract

**Background:**

Cognition and more recently social cognition, have been shown to be a strong predictor of clinical and functional outcome in psychosis. Jumping to Conclusions (JTC), which is defined as the proneness to require less information before forming beliefs or making a decision, has been related to the formation and maintenance of delusions. However, its relevance to longer-term outcome is unclear. On the other hand, there is evidence in the literature to suggest differences of patterns in clinical outcome and service based ethnicity. Using data from the GAP case-control study of first-episode psychosis (FEP), we set out to test whether the premorbid IQ, educational level and presence of JTC would predict poor clinical outcome at 4 year controlling for ethnicity.

**Methods:**

431 FEP patients were assessed with the positive and negative syndrome scale (PANSS) and Global Assessment of Functioning (GAF). Premorbid IQ was measured by the National Adult Reading Test (NART) scale, probabilistic reasoning “Beads” task was applied and educational levels were recorded alongside with socio-occupational variables at the time of recruitment. Follow-up data over an average period of 4 years were obtained from the electronic psychiatric clinical records in the South London and Maudsley NHS Foundation Trust (SLaM); including items concerning clinical course and outcomes (remission, intervention of police, use of involuntary treatment – the Mental Health Act (MHA) -, and inpatient days). We build different regression models using separately premorbid IQ, education level and JTC as predictors for each clinical outcome, both unadjusted and adjusted by ethnicity, age and gender.

**Results:**

Higher educational level was predictor of clinical remission [adjusted OR=1.9, 95% confidence interval (CI) 1.2–3, p=0.005]. FEP who presented JTC at baseline were more likely during the follow up period to be detained under the MHA [adjusted OR=11.23, 95% confidence interval (CI) 2.64–47.76, p=0.001], require intervention by the police (adjusted OR=10.76, 95% CI 2.4–48.26, p=0.002) and have longer admissions (adjusted IRR=4.04, 95% CI 1.43–11.36, p=0.008). We couldn’t find any predictor effect for clinical outcome for premorbid IQ. The association with level of education and JTC was not accounted for by socio-demographic variables including ethnicity.

**Discussion:**

Although we did not find association with premorbid IQ, educational level as indirect proxy of neurocognition showed a predictor effect for clinical remission. JTC in FEP is associated with serious subsequent consequences in terms of social disturbance and a poor therapeutic alliance. Our findings raise the question of whether the implementation of specific interventions to reduce JTC, such as Metacognition Training, may be a useful addition in early psychosis intervention programs.

